# Avoidance habit learning in adolescents and young women with anorexia nervosa: an fMRI study

**DOI:** 10.1111/jcpp.70019

**Published:** 2025-07-31

**Authors:** Julius Hennig, Ilka Boehm, Katharina Zwosta, Joseph A. King, Daniel Geisler, Hannes Ruge, Maria Seidel, Fabio Bernardoni, Inger Hellerhoff, Arne Doose, Sophie Pauligk, Henri Leschzinski, Veit Roessner, Uta Wolfensteller, Stefan Ehrlich

**Affiliations:** ^1^ Translational Developmental Neuroscience Section, Division of Psychological and Social Medicine and Developmental Neurosciences, Faculty of Medicine Technische Universität Dresden Dresden Germany; ^2^ Faculty of Psychology Technische Universität Dresden Dresden Germany; ^3^ Department of Child and Adolescent Psychiatry, Faculty of Medicine Technische Universität Dresden Dresden Germany; ^4^ Eating Disorders Research and Treatment Center, Department of Child and Adolescent Psychiatry, Faculty of Medicine Technische Universität Dresden Dresden Germany

**Keywords:** Adolescence, neuroimaging, habitual behavior, avoidance, eating disorder

## Abstract

**Background:**

Anorexia nervosa (AN), often with an onset in adolescence, is a complex eating disorder characterized by distorted body image, fear of weight gain, and extreme food restriction, leading to severe underweight. Excessive goal pursuit and avoidance behaviors have been proposed as key factors in AN, which over time may become over‐trained into habits.

**Methods:**

This study investigated the behavioral and neural correlates of habit learning in AN with an experiment consisting of three consecutive phases: (1) training goal‐directed behavior, (2) avoidance learning, and (3) a habit test. Forty‐five acutely underweight adolescent female patients with AN and 45 age‐matched healthy control participants underwent an fMRI scan.

**Results:**

No behavioral group differences were evident either during learning of avoidance habits or when testing habit strength. Importantly, however, the AN group showed both generally superior task performance and increased involvement of the frontoparietal brain regions during habit learning.

**Conclusions:**

Collectively, our findings provide novel evidence suggesting that excessive goal pursuit may predominate in young AN in an avoidance learning context. Future research should examine if this tendency develops into habit learning over time and investigate the speed and strength of avoidance habit formation in adults with a longer history of AN to further elucidate the intricate dynamic between goal‐directed and habitual processes in the disorder.

## Introduction

Anorexia nervosa (AN) is an eating disorder (ED) that typically develops in adolescence and is characterized by a distorted body image, fear of gaining weight, and extreme restriction of food consumption resulting in severe underweight (American Psychiatric Association, 2013). Popular theoretical perspectives propose excessive cognitive control and avoidance behaviors as key contributing factors (e.g. Fairburn, Shafran, & Cooper, 1999; Haynos, Widge, Anderson, & Redish, 2022; Melles, Spix, & Jansen, 2021). Another prominent view emphasizes that while such behaviors may be initially rewarding and goal‐directed, they become overtrained into maladaptive habits as illness duration progresses (O'Hara, Campbell, & Schmidt, 2015; Steinglass & Walsh, 2006; Uniacke, Timothy Walsh, Foerde, & Steinglass, 2018).

Habits are generally defined as learned behavior that over time becomes a relatively automatic response to specific cues, independent of the outcome (Wood & Rünger, 2016). Initially, goal‐directed behavior relies on response‐outcome (R‐O) associations, but with repetition, stimulus–response (S‐R) associations may become overtrained and insensitive to reinforcement or devaluation (Graybiel, 2008; Wood & Rünger, 2016). Importantly, such a shift from goal‐directed to habitual behavior has some advantages, as it requires no conscious effort and frees up cognitive capacities (Haith & Krakauer, 2018). Many daily behaviors, including food intake, become habitual to varying degrees (Wood & Rünger, 2016).

Maladaptive AN‐related behaviors such as food avoidance and overexercising have been shown to be rigid and persistent, and thus resistant to treatment (Coniglio, Cooper, & Selby, 2022; Mayer, Schebendach, Bodell, Shingleton, & Walsh, 2012; Schebendach, Mayer, Devlin, Attia, & Walsh, 2012). Several questions arise from the notion that such behaviors may reflect maladaptive habits (Steinglass & Walsh, 2006; Walsh, 2013). For example: (1) is habit learning altered (e.g. faster) in individuals with AN; (2) are newly acquired habits stronger and more resistant to change in AN; (3) are differences in (1) and (2) present in domains unspecific to EDs? To date, studies investigating habitual behavior in AN are sparse (Uniacke et al., 2018). Among these, some have used questionnaires such as the Self‐Report Habit Index (SRHI; Verplanken & Orbell, 2003) and found that patients with AN showed higher eating‐related habit strength compared to healthy controls (HC) (Schröder, Danner, Steinglass, & Foerde, 2024; Steinglass et al., 2018) and that food restriction was best explained by habit strength (Coniglio et al., 2017). Importantly, habit strength seems to be correlated with illness duration and symptom severity in AN (Davis, Walsh, Schebendach, Glasofer, & Steinglass, 2020). Using ecological momentary assessment (EMA), a method that samples behavior and experiences in real‐time in everyday life, we previously found that patients with AN exhibit more habits compared to HCs, both in ED‐related and ED‐unrelated categories (Seidel et al., 2022). Experimental support has been mixed; however, it has mostly focused on approach‐related habits (aimed at obtaining rewards; Foerde, 2025). For example, while Favier et al. (2020) showed convergent evidence of enhanced approach‐related habit formation, both Godier et al. (2016) and Westwater et al. (2022) found no behavioral differences in an outcome‐devaluation task. Notably, Godier et al. (2016) also reported no group differences in avoidance‐related habit learning (aimed at avoiding losses or other aversive outcomes). Potential reasons for these disparities may include age/developmental effects and duration of illness in the studied samples. Given that the majority of previous research has been in adults, a better understanding of habit learning in younger, non‐chronic individuals with AN is needed.

To date, only very few studies have examined the neural basis of habitual behavior in AN. In a previous fMRI study (Steding et al., 2019), we identified a subgroup of participants with AN who displayed habit‐like behavior during a reward learning task and a decreased BOLD signal in the medial orbitofrontal cortex compared to the subgroup showing more goal‐directed behavior. Foerde, Steinglass, Shohamy, and Walsh (2015) reported that patients with AN activated the dorsal striatum when making food choices and thus suggested that persistent food avoidance may be habitualized in adults with AN (but not adolescents, see Lloyd et al., 2024). In a resting‐state functional connectivity study, Haynos et al. (2019) observed disturbances in connectivity in reward and habit circuits (nucleus accumbens and the ventral and dorsal caudate) in older adolescents and young adults with AN. However, none of these neuroimaging studies employed tasks specifically designed to target habitual behavior and therefore could only make indirect inferences based on habit‐related brain regions and habit‐like behavior. Given fMRI findings suggestive of abnormally elevated cognitive control in AN (Steward, Menchon, Jiménez‐Murcia, Soriano‐Mas, & Fernandez‐Aranda, 2018), behavioral manifestations in AN might not merely arise from an isolated dominance of the habitual or the goal‐directed system. Rather, the interplay and balance between these two systems could be pivotal (Foerde et al., 2021) and neuroimaging methods might be particularly useful to shed light on this interaction.

The current fMRI study examined whether habit learning and avoidance behavior might be altered already in adolescents and young women with acute AN and a relatively short duration of illness, compared to carefully age‐matched HC. To this end, we adapted an established habit task that was validated for use in an MRI environment (Wang et al., 2024; Zwosta, Ruge, Goschke, & Wolfensteller, 2018). In our version, we implemented negative reinforcement to target avoidance behavior, a mechanism closely linked to restrictive eating, high punishment sensitivity, and harm avoidance in AN (Foerde, 2025; Haynos, Lavender, Nelson, Crow, & Peterson, 2020). Importantly, this task has been shown to be sensitive to the process of habit learning and its underlying neural correlates. We expected stronger behavioral avoidance habit effects in the AN group. Additionally, we hypothesized that there would be alterations in training‐related neural responses during avoidance habit learning. These alterations were anticipated in habit‐related regions, such as the basal ganglia, as well as regions associated with goal‐directed behavior and cognitive control, including the prefrontal and parietal cortices (Dolan & Dayan, 2013). Lastly, we integrated the aforementioned EMA data on habit frequencies (Seidel et al., 2022) as a real‐life measure to inform the analysis of the fMRI data.

## Methods

### Sample description

We collected data from 136 female participants, including 51 individuals diagnosed with acute AN according to DSM‐5 and 85 HC participants. After conducting quality control (QC) checks on the neuroimaging and behavioral data of the 51 patients with AN, six individuals had to be excluded (four after neuroimaging QC due to poor image quality and two after behavioral QC due to poor performance, see Appendix S1) leaving a total of 45 patients with AN. To account for potential developmental effects and optimize comparisons between groups, we implemented a pairwise age‐matching algorithm (Munkres, 1957) to minimize age differences, resulting in a final sample of 90 individuals, including 45 HC participants (age range: 12.9–24.0 years) age‐matched to 45 patients with AN (age range: 13.0–24.1 years) (Appendix S1.2).

All patients were admitted to eating disorder programs of a university child and adolescent psychiatry or psychosomatic medicine department and were assessed within 96 h after beginning a behaviorally‐oriented nutritional rehabilitation program. HC participants were recruited through advertisement among middle school, high school, and university students and had to be of normal weight, eumenorrhoeic, and without any history of psychiatric illness.

We applied several additional exclusion criteria for each group, including, most importantly, psychotropic medication within 4 weeks prior to the study, binge eating, or diagnosis of bulimia nervosa, substance use disorder, neurologic, or medical conditions (psychiatric diagnoses were based on DSM‐IV criteria; for further details regarding the diagnostic procedure, see Appendix S1.2). Importantly, HC participants were excluded if they had any psychiatric disorder, while participants with AN were allowed to show depressive and anxiety symptoms.

### Clinical and questionnaire measures

For all participants, current diagnoses of EDs were ascertained by evaluation of the expert form of the Structured Interview of Anorexia Nervosa and Bulimia Nervosa (SIAB‐EX; Fichter & Quadflieg, 1999), which was adapted to DSM‐5 criteria. Additionally, the Mini‐International Neuropsychiatric Interview (Lecrubier et al., 2010) was used to diagnose active psychiatric disorders. Interviews were conducted by clinically experienced and trained research assistants under the supervision of the attending child and adolescent psychiatrist.

In addition to the clinical interviews, we administered questionnaires to characterize our sample and facilitate further statistical analyses (e.g. correlations with fMRI results). ED‐related symptoms were assessed with the Eating Disorders Inventory‐2 (EDI‐2; Paul & Thiel, 2005) and general psychopathology with the Symptom Checklist‐90‐R (SCL‐90‐R; Franke & Derogatis, 2002). Depressive symptoms, which often co‐occur with AN symptoms, were examined using the Beck Depression Inventory II (BDI‐II; Hautzinger, Kühner, & Keller, 2009). To account for developmental factors, BMI standard deviation scores (BMI‐SDS) were calculated for each participant to adjust for age and sex (Kromeyer‐Hauschild et al., 2001). We used the Brief Self‐Control Scale (BSCS; Sproesser, Strohbach, Schupp, & Renner, 2011) to measure trait self‐control, given its potential relevance to cognitive control processes. The Behavioral Inhibition System/Behavioral Activation System (BIS/BAS; Strobel, Beauducel, Debener, & Brocke, 2001) was used to assess motivation to approach rewarding and avoid aversive outcomes such as punishment. Lastly, we collected EMA data regarding the frequency of food and hygiene habits over the course of 7 days, with eight prompts per day using a smartphone app, providing real‐world context to the laboratory findings (as described in Seidel et al., 2022; Appendix S1.3).

### Procedure/habit‐goal task

**Figure 1:**
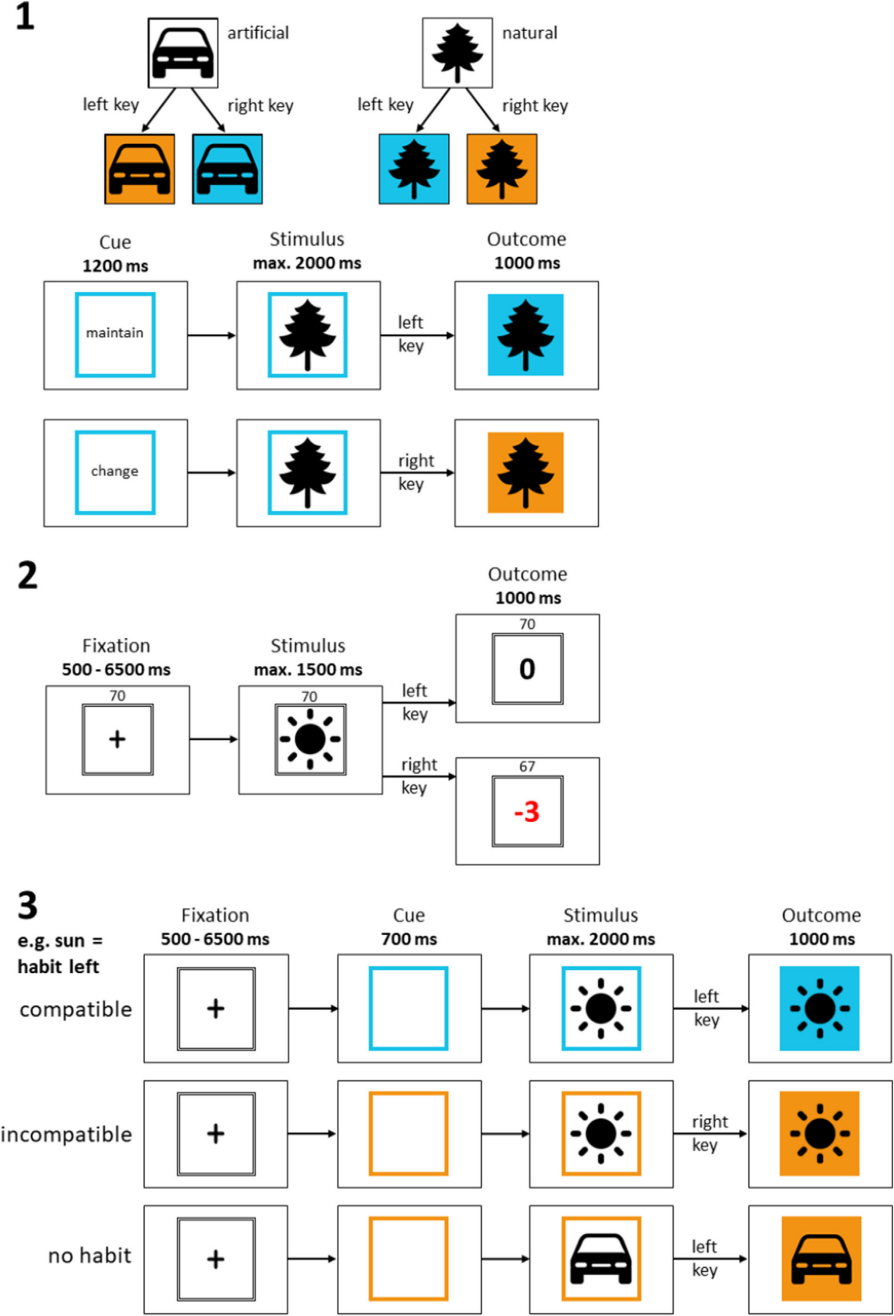
Experimental avoidance habit learning paradigm consisting of three consecutive phases (phases 2 and 3 inside an MRI scanner). (1) Phase 1: Goal‐directed behavior was established by presenting hierarchical response‐outcome associations using 2 color cues (orange, blue) and 2 stimulus categories (3 ‘artificial’ and 3 ‘natural’ stimuli). Additionally, the trials consisted of a ‘maintain’ or ‘change’ cue in order to prevent habit learning. (2) Phase 2: Trials involving punishment were used to train habitual avoidance behavior through extensive training of S‐R‐O associations, which had to be identified by trial and error. (3) Phase 3: The overtrained stimulus–response associations of phase 2 were put into competition with goal‐directed behavior to test individual habit strength using the contingencies learned in phase 1 (without the ‘maintain’ and ‘change’ cue). Trials could be compatible when the correct response based on the color cue and category matched the response learned in phase 2 for a certain stimulus and incompatible when they did not match. Additionally, ‘no habit’ trials were based on the stimuli that were not used in phase 2

The habit task (Zwosta et al., 2018) employed in this study consisted of three consecutive phases and was executed with E‐Prime 2.0 (Psychology Software Tools, Pittsburgh, PA); see Figure 1 and Figure S4 for additional details. The first phase was performed outside of the MRI scanner. The latter two were performed inside the MRI scanner. In order to make the study more feasible for our young and severely ill sample, we modified the original task by Zwosta et al. (2018) in the following ways: (1) of the original two conditions, ‘reward’ and ‘punishment’ in phase 2, we only used the punishment condition to focus on avoidance learning; (2) we reduced the number of stimuli (3 artificial and 3 natural) and consequentially the total number of trials in each phase; and (3) we replaced stimuli associated with food (mushroom, cow) to prevent an adverse reaction by the AN group. To this end, we used stimuli depicting a sun, a tree, and a flower for the natural category and a scissor, a car, and a ball for the artificial category.

Throughout the task, participants received standardized written instructions explaining the rules for each phase.

#### Phase 1: Establishing goal‐directed behavior (132 trials)

This phase was intended to establish goal‐directed behavior using hierarchical response‐outcome associations. Participants were instructed to maintain or switch the outcome color depending on the stimulus category (artificial or natural with three stimuli each), which was associated with a left or right button press. In detail, each trial started with a ‘maintain’ or ‘switch’ cue, framed by a blue or orange colored square. This cue was then replaced by one of the six stimuli, and participants then pressed a button to switch or maintain the current outcome color based on the stimulus category. The stimulus background then changed to the respective outcome color. The word ‘Error’ was displayed below the square for incorrect responses, and the trial was repeated until the response was correct.

#### Phase 2: Avoidance habit learning (392 trials)

This phase aimed to establish avoidance habits by overtraining certain S–R associations as participants tried to avoid punishment for incorrect responses. Negative feedback was given as deductions from total earnings over seven task blocks of 56 trials each. Two of the three stimuli from both categories were reused, with a randomly selected 2 × 2 set, each presented in 98 trials. At the beginning, participants were informed that the categories were now irrelevant and that they had to determine the correct left or right response for each of the four stimuli through trial‐and‐error. Of both categories, one stimulus was associated with a right response and one with a left response.

Each trial commenced with a fixation cross within a black‐and‐white lined square, followed by the presentation of a stimulus. Participants received immediate feedback on their point outcome after responding. The total number of points accumulated during the current task block was displayed at the top of the screen.

Participants started each block with 70 points, losing 3 points for each incorrect response while correct responses did not change the point total. Each of the 7 × 70 points equaled 0.01€, allowing the participants to gain a maximum of 4.90€ (additionally to a general compensation for their participation).

#### Phase 3: Testing habit strength (112 trials)

This phase was intended to test habit strength by putting the goal‐directed behavior established in phase 1 (without the ‘switch’ and ‘maintain’ cues) into competition with the habitual avoidance behavior trained in phase 2. Participants were informed that they would no longer lose points, removing the S‐R‐O contingency. Hence, any tendency to continue trained behavior indicated habitual responding rather than being motivated by avoiding loss of points.

In detail, each trial began with a fixation cross, followed by a colored frame (blue or orange) and one of the six stimuli at the center of the frame. Based on the R‐O contingencies of phase 1, trials were either habit‐compatible or habit‐incompatible. In compatible trials, the required goal‐directed response matched the habitual response from phase 2, while in an incompatible trial, it opposed the habitual response. As all six stimuli were used in phase 3, four stimuli were associated with a trained habit while two stimuli were not (referred to as ‘no habit’ condition).

### Structural and functional image acquisition and data processing

Images were acquired using standard sequences with a 3 T whole‐body MRI scanner (TRIO; Siemens, Erlangen, Germany) equipped with a standard head coil (Appendix S1.5). Functional and structural images were processed with SPM12 (http://www.fil.ion.ucl.ac.uk/spm/software/spm12/) within the Nipype framework (http://nipy.sourceforge.net/nipype/; Gorgolewski, Storkey, Bastin, & Pernet, 2012) following standard procedures (Appendix S1.6). We evaluated fMRI data quality by manual inspection and using the artifact detection tool (ART; Whitfield‐Gabrieli et al., 2009). Volumes that exceed an intensity threshold of three standard deviations or a threshold of 2 mm normalized movement in any direction were classified as outliers and excluded from statistical models (motion‐outlier: AN: 0.76 ± 2.38; HC: 0.71 ± 1.71; intensity‐outlier: AN: 8.91 ± 4.45; HC: 9.00 ± 4.22; both n. s. with *p* > .90).

### Statistical analyses

Demographic, clinical, and behavioral task‐based data were analyzed using SPSS (IBM SPSS Statistics 28.0.0.0).

The behavioral metrics of interest were error rate (ER) and reaction times (RT) in all three phases. To verify that the participants start to show goal‐directed responses after initial orientation with the task, we grouped the trials of phase 1 into four blocks containing 33 trials each. We conducted an analysis of variance (ANOVA) incorporating blocks and the ‘switching’ cue (maintain vs. switch) as within‐subject factors, with group as the between‐subject factor. This was done to examine potential learning effects, which would be indicated by a decrease in ER. For phase 2, we ran ANOVAs for ER and RT with training (block 1–7) as within‐subject factor and group as between‐subject factor. For phase 3, we computed ANOVAs for ER and RT in the goal‐directed trials with compatibility (compatible vs. incompatible) as within‐subject factor and group as between‐subject factor to validate our experimental approach. Additionally, for phase 3, we computed a ‘habit‐driven compatibility effect’ metric for both RT and ER by subtracting the values of the incompatible condition from the compatible condition. The more negative the value, the stronger the habit. We used this measure to explore the association between training‐related changes in neural activity during habit learning in phase 2 and the behavioral habit‐driven compatibility effect in phase 3.

Regarding the fMRI data, on the single‐subject level in phase 2, we computed a linear parametric regressor set to the cumulative number of correct responses to model the training‐related hemodynamic response change using an event‐related design with events locked to the stimulus onset. Correct, incorrect, and missed trials, as well as the six motion parameters (for each motion and intensity outlier volume) were also modeled as non‐parametric nuisance regressors.

Our second‐level fMRI analyses focused on phase 2 of the experiment. We first ran whole‐brain analyses for all participants using the training regressor to verify general avoidance habit learning effects as in Zwosta et al. (2018). Afterwards, we ran the same whole‐brain analyses comparing the two groups for differences in avoidance habit learning using a two‐sample t test. To control for false positives, all analyses were family‐wise error (FWE) corrected using 3DClustSim (https://afni.nimh.nih.gov/, version from 24 Aug 2018). At a voxel‐wise threshold of *p* < .001, cluster size had to exceed 34.8 voxels to be considered significant at a combined threshold of *p* < .05 (FWE).

For post‐hoc analyses, we extracted the β values averaged from all voxels of the significant clusters using SPM 12 for each participant and used SPSS for further analyses.

To test whether regions with group differences interact as part of a network, we conducted exploratory generalized psycho‐physiological interaction (gPPI) analyses. These analyses aimed to determine how the significant group effect clusters identified in phase 2 interact with each other. The gPPI analyses were performed using the gPPI toolbox (McLaren, Ries, Xu, & Johnson, 2012; Appendix S1.7) applying the same training and group regressors as above. Seed regions were based on significant group effect clusters of phase 2 and limited to other significant clusters. More specifically, for each group effect cluster, we created a 5 mm sphere surrounding its peak voxel. In order to avoid the problem of multiple comparisons, we limited the number of gPPIs to hypothesis‐relevant clusters in regions associated with habitual behavior and cognitive control.

Lastly, we used ecological momentary assessment (EMA) data previously published in Seidel et al. (2022) that was collected from 38 of our 45 patients with AN and 27 of our 45 HC participants as part of our greater ongoing study of AN (for more details regarding the EMA assessment, see SM 3) to explore potential relationships between the fMRI activation in regions in which group differences were observed and real‐life habit measures in the AN group (by conducting correlation analyses of extracted β values). This EMA data focused on habitual behavior in the daily life of our participants (Seidel et al., 2022): the frequency of ED‐related (eating) and ED‐unrelated (hygiene) habits. In detail, we used the cumulative number of reported habits over the course of 7 days (corrected for the compliance), which Seidel et al. found to be increased significantly in the AN group compared to the HC group for both categories.

## Results

### Demographic data and clinical measures

As expected, there were no age differences, but participants with AN had lower BMI‐SDS and higher EDI‐2, SCL‐90‐R, and BDI‐II scores (Table 1). Additionally, we found no group differences in the BSCS total score (but see SM 2 for group differences in ‘self‐discipline’ with AN>HC) or BAS, while the AN group showed higher BIS values indicating elevated punishment sensitivity in AN. IQ estimates were lower in AN and therefore accounted for in sensitivity analyses of all hypothesis‐relevant group comparisons (Appendix S1.11, Tables S6–S9). Regarding everyday habits, EMA measures suggested significantly more habits in both categories, confirming that self‐reported habit frequency is increased in AN (Seidel et al., 2022) as expected (given the partial sample overlap).

**Table 1:** Demographic and clinical characteristics of the sample

	AN		HC		*t*	*p*	*d*
	*M*	*SD*	*M*	*SD*			
Age	16.14	2.15	16.20	2.10	0.15	.882	0.03
Ethnicity	European: *n* = 44		European: *n* = 44				
	Asian: *n* = 1		Siberian: *n* = 1				
Socioeconomic status	3.67	0.78	3.74	0.91	0.38	.707	0.08
Time since onset of AN (months)	13.86	12.83	–	–		–	–
BMI‐SDS	−3.41	1.19	0.55	0.66	17.10	<.001	3.60
IQ	107.21	10.44	116.68	9.65	4.37	<.001	0.94
EDI2 total	218.75	49.22	132.87	20.12	10.50	<.001	2.30
SCL‐90‐R GSI	1.15	0.66	0.21	0.16	9.03	<.001	1.97
BDI‐II	25.52	10.63	4.06	4.21	12.45	<.001	2.65
BSCS total	44.21	8.40	43.82	7.67	0.23	.821	0.05
BIS	22.76	3.08	19.78	2.36	5.10	<.001	1.09
BAS	40.57	3.82	39.42	4.28	1.32	.191	0.28
Frequency food habits (EMA)	0.415	0.28	0.176	0.13	4.56	<.001	1.03
Frequency hygiene habits (EMA)	0.467	0.30	0.241	0.16	3.98	<.001	0.89

AN, participants with acute anorexia nervosa; HC, healthy control participants. Group differences were tested using two‐sided Student's *t*‐tests. IQ, Intelligence Quotient based on a short version of the German adaption of the Wechsler Adult Intelligence Scale (von Aster, Neubauer, & Horn, 2006), BMI‐SDS, body mass index standard deviation score; SCL‐90‐R GSI, Symptom Checklist‐90‐R Global Severity Index; BDI‐II, Beck's Depression Inventory‐II; EDI‐2, Eating Disorder Inventory‐2; BSCS, Brief Self‐Control Scale; BIS, Behavioral Inhibition System; BAS, Behavioral Activation System; EMA, ecological momentary assessment. As a measure of effect size, we included Cohen's *d*. Of the 45 patients with AN, *n* = 37 were of the restrictive and *n* = 8 of the binge/purge subtype; additionally, *n* = 9 reported comorbid psychiatric disorders (*n* = 4 depressive disorder, *n* = 2 obsessive compulsive disorder, *n* = 1 panic disorder, *n* = 3 social phobia and *n* = 1 anxiety disorder not otherwise specified). Socioeconomic status (SES) was assessed based on educational and occupational level, using a scale from 0 (lowest; no school qualification) to 5 (highest; university degree) (adapted from Ganzeboom, De Graaf, & Treiman, 1992). As most participants were adolescents still in education and living with their parents or guardians, parental SES was used as a proxy. For the three AN participants living independently, their own SES was used.

### Behavioral results

#### Results of phase 1: establishing goal‐directed behavior

Indicating that the R‐O associations were acquired successfully, a significant main effect of block showed that ER generally decreased in both groups during phase 1 (*F*(3, 264) = 31.56, *p* < .001). Notably, this effect was not visible in RTs (*F*(3, 264) = 0.03, *p* = .995). As expected, the main effect of switching indicated that ER was generally lower and RTs faster under the maintain condition (*F*(1, 88) = 237.49, *p* < .001; *F*(1, 88) = 8.14, *p* = .005). Additionally, indicating that the AN group acquired R‐O associations more efficiently than HC, a significant group × block interaction showed that the decrease in ER across phase 1 was steeper in the AN group (*F*(3, 264) = 3.10, *p* = .027). See Figure S1 and Table S2 in Appendix S1.8 for more details.

#### Results of phase 2: avoidance habit learning

**Figure 2:**
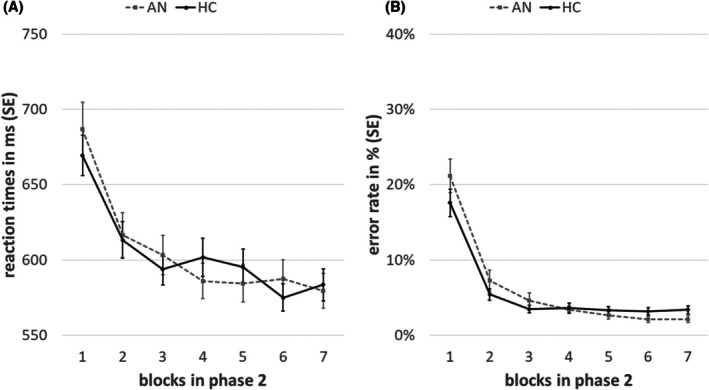
Behavioral results of phase 2. Reaction times (A) and error rates (B) over the course of phase 2 (training), separated for the acute anorexia nervosa (AN) and the healthy control (HC) group. Error bars depict the standard error (SE) of the mean

Confirming that the overtraining of phase 2 was successful, significant main effects of training were evident both in RTs and ER, indicating faster and more accurate performance over the course of the seven blocks (ER: *F*(6, 528) = 94.9, *p* < .001; RT: *F*(6, 528) = 47.55, *p* < .001). There were no general group differences nor group × training interactions (all *F* < 2.04 and all *p* > .05; Figure 2).

#### Results of phase 3: Testing habits

In the critical behavioral test of habits, expected main effects of compatibility were evident in RT, but not in ER (Table 2 and Figure S2, Appendix S1.9). As for group differences, a main effect of group was revealed in ER (but not in RT) indicating generally higher accuracy in AN over all compatibility conditions. No group × compatibility interactions were significant in RT or ER.

**Table 2:** Repeated‐measures ANOVA of the behavioral data of phase 3

	RT				ER			
	*df*	*F*	*p*	η_partial_	*df*	*F*	*p*	η_partial_
Compatibility	1, 88	10.01	.002	0.10	1, 88	0.93	.338	0.01
Group	1, 88	0.13	.724	<0.01	1, 88	5.54	.021	0.06
Compatibility × group	1, 88	1.87	.175	0.02	1, 88	0.48	.488	0.01

ER, error rate; RT, reaction times.

### Neuroimaging results

**Figure 3:**
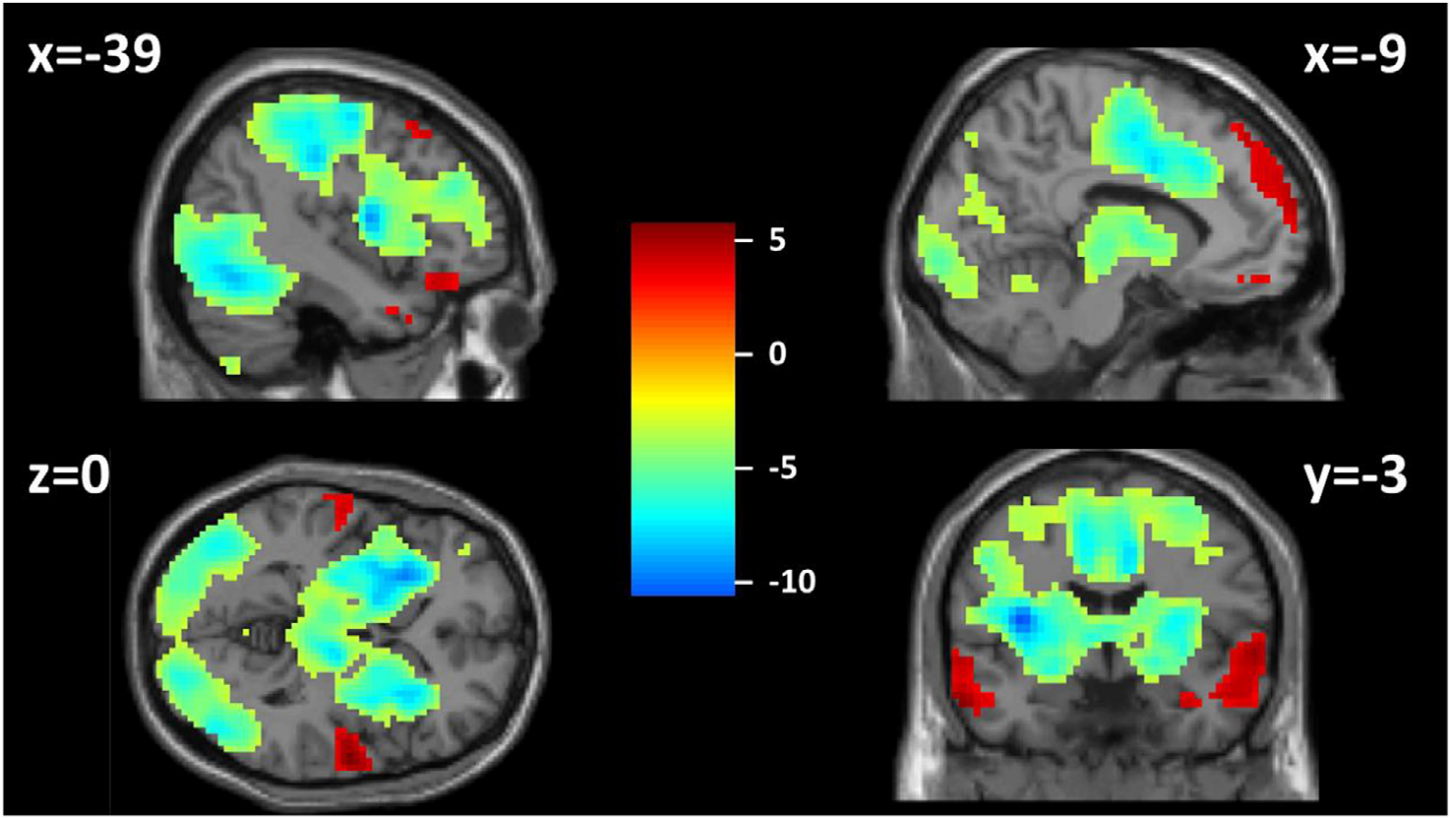
Neuroimaging results of the main effect of training in phase 2. Brain regions showing an increase (red) and decrease (blue) of activity during training for all participants. *T* map, FWE *p* < .05

**Figure 4:**
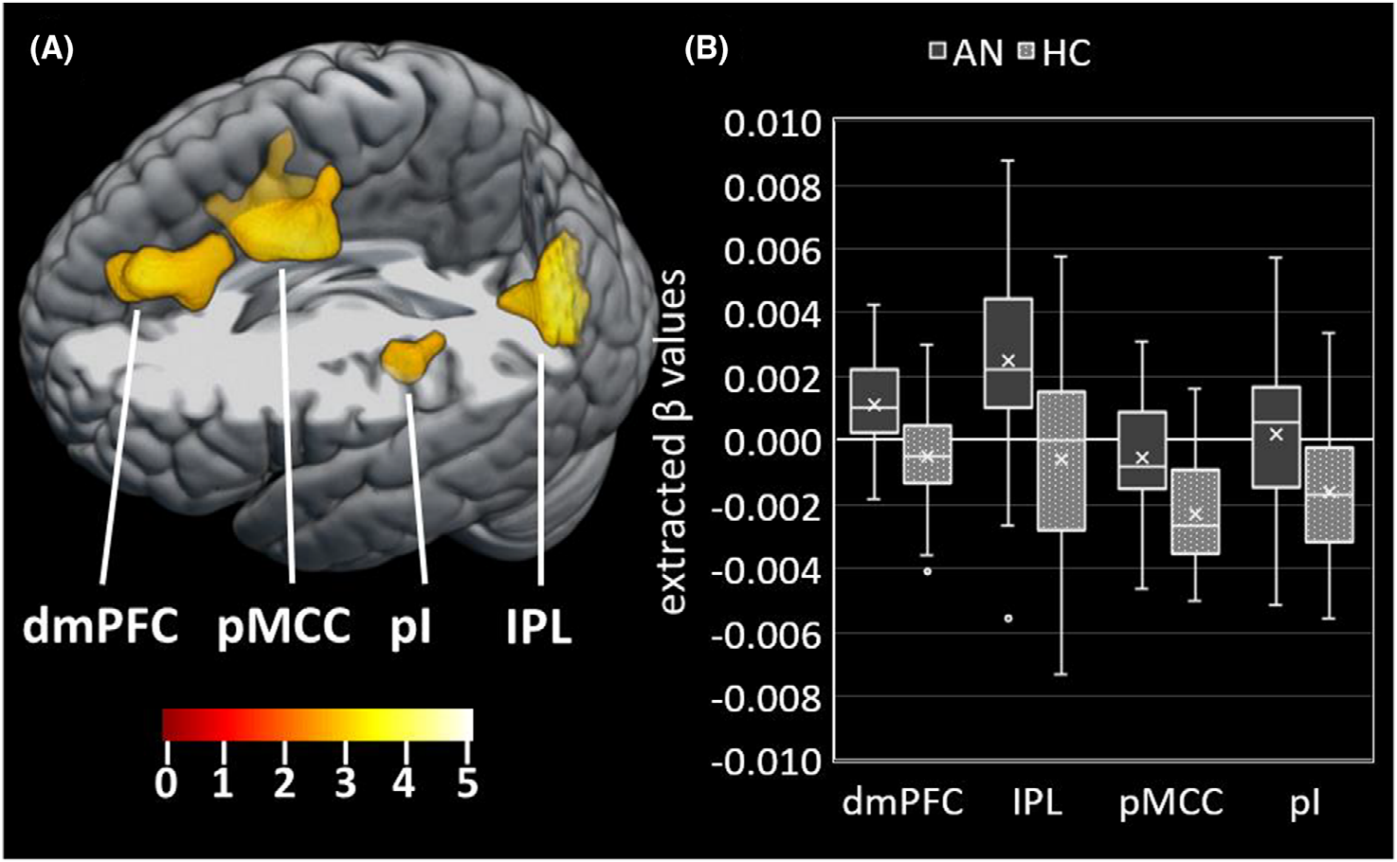
(A) Clusters showing the training‐related group differences in phase 2 (*t*‐test). Clusters in the dorsomedial prefrontal cortex (dmPFC) and the inferior parietal lobe (IPL) showed a stronger increase of neural activity for the acute anorexia nervosa (AN) group compared to the healthy control (HC) group. Clusters in the posterior midcingulate cortex (pMCC) and the posterior insula (pI) showed a stronger decrease of neural activity for the HC compared to the AN group. *T* maps, FWE‐corrected *p* < .05. (B) Boxplot of the extracted mean β beta values of the BOLD signal slopes in the PFC, IPL, pMCC, and pI for the AN and the HC groups. The horizontal line depicts the group median, and the cross depicts the group mean

Main effects of task indicating significant training‐related activation were confirmed in several brain regions (FWE‐corrected *p* < .05; see Figure 3 and Table S5, Appendix S1.10) largely aligning with Zwosta et al. (2018). More importantly, significant group differences in training‐related changes indicating higher BOLD signal slopes in AN compared to HC (Figure 4, Table 3) were evident in four clusters that partially overlapped regions identified in the main effects analysis. Two were located in a dorsomedial region of prefrontal cortex (dmPFC) and the inferior parietal lobe (IPL), both showing an overlap with the frontoparietal control network (FPN), and two were located in the posterior mid‐cingulate cortex (pMCC) and the posterior insula (pI). More specifically, group comparisons of extracted mean β values indicated that participants with AN showed a training‐related activation increase in the dmPFC and the IPL, while the HC group showed no change in activation. In contrast, HC participants showed a significant training‐related activation decrease in pMCC and the pI, while the AN group showed no notable change (Figure 4). A sensitivity analysis covarying for the individual change in RT across training showed that phase 2 group differences were robust (Appendix S1.11).

**Table 3:** Whole‐brain results of the training‐related group differences in phase 2. FWE‐corrected at *p* < .05

Direction	Region of peak voxel	Peak voxel MNI coordinates			*T*_max_	Cluster size
AN > HC	Dorsomedial prefrontal cortex (left)	−15	42	42	4.54	186
		−15	30	35	4.41	
		−3	45	35	3.82	
	Inferior parietal lobe (left)	−51	−66	42	4.73	156
		−36	−57	31	4.18	
	Posterior midcingulate cortex	−9	−3	38	5.20	324
		9	−6	35	5.03	
		0	−15	50	3.73	
	Posterior insula (left)	−42	−24	19	3.74	44
HC > AN	–	–	–	–	–	–

AN, patients suffering from acute anorexia nervosa; HC, healthy control participants; MNI coordinates, Montreal Neurological Institute coordinates; *T*_max_ = peak intensity *t* value.

To further explore the connectivity dynamics over the course of the habit training, we conducted gPPI connectivity analyses using the two FPN clusters (dmPFC and IPL) and the pMCC (motor response; Vogt, 2016) as seed regions (Appendix S1.12). We then masked the results for each seed region using the respective three remaining clusters. Indicating a gradually increased functional coupling over the course of phase 2, this approach revealed overall training‐related increases in connectivity between the dmPFC and the IPL (*k* = 122 voxels) and vice versa (*k* = 10 voxels). We found no connectivity decreases between the four clusters and no group differences.

Exploratory Spearman correlation analyses investigated whether training‐related activation changes (mean β values) in the identified dmPFC, IPL, the pMCC, and pI regions during avoidance habit learning in phase 2 predicted behavioral habit‐driven compatibility effects in phase 3 in our AN sample, but no relationships reached significance (RT: all |*r*| < −.24 and all *p* > .108; ER: all |*r*| < .06 and all *p* > .676).

### Associations between behavioral and neuroimaging results with clinical measures

Exploratory analyses of potential relationships between the behavioral compatibility effects of phase 3 and dmPFC, IPL, pMCC, and the pI (extracted β values) activity during training in the AN group with clinical variables (BMI‐SDS, EDI‐2 total, BDI‐II, SCL‐90‐R total, IQ, age, duration of illness, BIS, BAS and BSCS) as well as the EMA measures of eating and hygiene habits revealed no significant associations (behavioral: all |*r*| < .25 and all *p*_FDR‐adjusted_ > .959; neuroimaging: all |*r*| < .40 and all *p*_FDR‐adjusted_ > .672).

## Discussion

To our knowledge, this was the first study to use an experimental paradigm (Zwosta et al., 2018) to investigate the neural correlates of avoidance habit learning in anorexia nervosa (AN). During fMRI, participants learned and extensively trained responses in order to train avoidance habits that were subsequently put into competition against previously established goal‐directed behavior. Behaviorally, we found no indication that the AN group differed from the HC group with respect to avoidance habit learning (phase 2) and the habit‐driven compatibility effect – which may be a proxy for habit strength (phase 3). However, contrary to our motivating hypothesis of stronger avoidance habits in AN, but reminiscent of excessive goal pursuit (Kaye, Wierenga, Bailer, Simmons, & Bischoff‐Grethe, 2013), patients showed overall superior general behavioral performance in experiment phases 1 (faster learning) and 3 (fewer errors) ‐ regardless of conditions (i.e. independent of habits). On the neural level, we observed an expected overall training‐related BOLD signal decrease in phase 2 (avoidance habit learning) in a wide range of brain regions similar to Zwosta et al. (2018). Most importantly, we found that during avoidance habit learning the AN group showed a training‐related activation increase in regions of the frontoparietal network (FPN) typically involved in cognitive control, attention, and goal‐directed behaviors (Nee, 2021), namely the dmPFC and the IPL, while the HC group did not. Together, the absence of group differences in behavioral measures of habit learning or fMRI activation in habit‐related brain regions (e.g. basal ganglia), but increased activity in regions associated with cognitive control hint at a potential imbalance between goal‐directed and habitual brain systems during avoidance learning in young patients with AN.

The lack of altered behavioral correlates of habits in AN observed in the current study contrasts with clinical observations (Coniglio et al., 2017; Davis et al., 2020; Seidel et al., 2022; Steinglass et al., 2018), but is consistent with previous experimental findings (Foerde et al., 2021; Godier et al., 2016; Westwater et al., 2022). This may reflect developmental differences in the goal‐directed and habitual systems (Decker, Otto, Daw, & Hartley, 2016), a relatively short illness duration in our young sample, or intact early habit learning in AN, at least for behaviors not directly tied to AN symptomatology. Relatedly, studies have shown associations between restrictive food choice and neural activity in habit‐related brain regions in adults with AN, but not in adolescents (Foerde et al., 2015; Lloyd et al., 2024). In the current study, the AN group's generally superior task performance may reflect extreme goal pursuit and overcontrol (Haynos et al., 2022). Fittingly, previous studies documented similarly enhanced performance in different cognitive domains (King et al., 2019; Lloyd, Yiend, Schmidt, & Tchanturia, 2014). This behavioral pattern also aligns with our findings of increased FPN activity in AN during avoidance habit learning as well as prior research that has shown brain activity alterations in AN in regions associated with cognitive control, even in tasks not explicitly targeting these functions. For example, Ehrlich et al. (2015) observed increased FPN activation during reward anticipation in an instrumental motivation task in weight‐recovered individuals with a history of AN. Elevated prefrontal activity in participants with AN has also been reported in food‐cue reversal learning tasks (Eddy et al., 2023; Hildebrandt et al., 2018). However, as our fMRI analyses did not account for RT as a regressor, and given that an overcontrol interpretation of the current results rests in part on reverse inference (Poldrack, 2006), other interpretations should therefore be considered.

One alternative interpretation of the group difference observed in the dmPFC and the IPL can be drawn based on the spatial overlap with the default mode network (DMN), a brain system active during rest and mind‐wandering (Raichle, 2015), as well as during active task conditions (Sormaz et al., 2018). Interestingly, the DMN's involvement has been termed ‘autopilot’ by some authors (Vatansever, Menon, & Stamatakis, 2017) and its activity has been observed to be related to the level of automaticity, a precursor of habitual behavior (Shamloo & Helie, 2016). In the context of our study, it could be speculated that AN individuals may switch into such a mode more quickly during habit learning, reflecting a precursory state of habitual behavior. This observation wouldn't necessarily contradict an overcontrol interpretation, as both processes are interlinked and since AN symptomatology seems to ‘challenge a simple goal‐directed‐versus‐habitual dichotomy’ (Foerde et al., 2021). Indeed, a clear distinction between the two processes has been questioned (Kruglanski & Szumowska, 2020) and controlled behavior can be habitualized (Galla & Duckworth, 2015).

Noteworthy group differences in training phase activation were also observed in the pMCC and pI in the current study. In both regions, the AN group displayed persistent activation while the HC group showed a relative activation decrease. Because both regions belong to somatosensory cortices involved in aversive processing (Vogt, 2016), our findings could indicate a difficulty in the AN group with adapting to the negative feedback in our experiment, aligning with previous fMRI findings in AN (Bär, Berger, Schwier, Wutzler, & Beissner, 2013; Bär, De La Cruz, Berger, Schultz, & Wagner, 2015; Bischoff‐Grethe et al., 2013; Frank, Collier, Shott, & O'Reilly, 2016; Strigo et al., 2013) as well as heightened punishment sensitivity and harm avoidance in AN (Haynos et al., 2020; Klump et al., 2000).

### Limitations

Several limitations of this study should be considered. First, there are challenges with experimental habit tasks, including ours (Foerde, 2025; Nebe, Kretzschmar, Brandt, & Tobler, 2024; Watson & de Wit, 2018). For example, the learning phase may need to be extended to train strong habits. Consequently, phase 3 of our task may have been too easy to detect subtle group differences in interference effects. Importantly though, a compatibility effect was evident in RTs, which may be a more reliable habit measure (Luque, Molinero, Watson, López, & Le Pelley, 2020). Furthermore, habit tasks depend on the interaction of habitual with goal‐directed behavior, making it challenging to isolate parameters solely related to habits (de Wit et al., 2018). Second, using negative reinforcement to train avoidance habits may differ mechanistically from approach‐related habits, necessitating more research to understand these differences, particularly given altered reward and punishment sensitivities in AN (Haynos et al., 2020). Third, habitual behavior in AN may be restricted to clinically relevant behavior in real life (as in Seidel et al., 2022) and difficult to capture in the laboratory. As a result, training avoidance habits using eating disorder‐unrelated stimuli (compared to e.g. food stimuli), as we have done here, may help prevent adverse (e.g. anxious) reactions in participants with AN, but might also obfuscate behavioral differences. Fourth, while we used EMA data as an additional source of information regarding habitual behavior, we did not include a corresponding classical questionnaire. Fifth, although our fMRI findings during habit learning are novel in AN, group differences were not linked to clinical or behavioral measures. Relatedly, our fMRI analyses did not account for RT, which can influence BOLD signal amplitude (Yarkoni, Barch, Gray, Conturo, & Braver, 2009). Therefore, interpretations remain tentative. Sixth, we did not examine pubertal status in our mostly adolescent sample (due to potential effects of the illness on pubertal development, Dempfle et al., 2013; and issues with self‐assessment validity, Desmangles, Lappe, Lipaczewski, & Haynatzki, 2006; Rasmussen et al., 2015). Lastly, given the intricate nature of goal‐directed and habitual behavior, future studies with larger and more diverse samples with varying durations of illness, as well as more sophisticated fMRI models, would be needed to replicate and extend our findings.

## Conclusion

Our findings suggest that the relationship between avoidance habit learning and AN may be more nuanced in younger individuals with a relatively short duration of illness. More specifically, they suggest that behaviors emblematic of AN, such as extreme dietary restrictions, over‐exercising, and a preoccupation with weight, might be initially driven by overcontrol (Baudinet et al., 2020; Haynos et al., 2022), but may only develop into habits after extensive repetition and disease chronification (Foerde et al., 2015; E. C. Lloyd et al., 2024). Future studies will need to further investigate habitual and goal‐directed behavior in individuals with longer durations of illness to better understand repetitive maladaptive behaviors in AN and their clinical relevance.

## Ethical considerations

This study was approved by the ethics committee of the Technische Universität Dresden (EK 536122015) and carried out in accordance with the latest version of the Declaration of Helsinki, and all participants (and their guardians if underage) gave written informed consent.

**Key points**

- While clinical observations suggest that habitual behavior plays a significant role in the development and maintenance of anorexia nervosa (AN), experimental evidence is scarce.
- Behaviorally, no avoidance habit‐related group differences between the AN and healthy control sample were evident. However, the AN group showed overall superior task performance.
- Together with our neuroimaging results, these findings suggest that overcontrol may play a significant role during avoidance habit learning in young AN.
- This adds new evidence to the notion of altered habit learning in AN, suggesting a more intricate dynamic between goal‐directed and habitual processes in the disorder.
- From a clinical perspective, our study underscores the need for more comprehensive therapeutic strategies that address both goal‐directed and habitual behavior.

## Supporting information


**Appendix S1.** Behavioral data quality control.
**Appendix S2.** Data collection and exclusion criteria.
**Table S1.** *t*‐Tests for the comparisons of the HCs included in current study with the ‘remaining’ HCs regarding demographic and clinical variables.
**Appendix S3.** EMA procedure and data.
**Appendix S4.** Experimental procedure (additional information).
**Appendix S5.** Structural and functional image acquisition.
**Appendix S6.** Functional image data processing and analysis.
**Appendix S7.** gPPI analysis.
**Appendix S8** and **Figure S1.** Behavioral results of phase 1.
**Table S2.** Repeated‐measures ANOVA of the behavioral data in phase 1.
**Appendix S9.** Behavioral results of phase 3, strategies and sensitivity analysis.
**Figure S2.** Behavioral results of phase 3.
**Table S3.** Repeated‐measures ANOVA of the inverse efficiency score of the behavioral data of phase 3.
**Table S4.** Repeated‐measures ANCOVA of the behavioral data of phase 3 with phase 1 mean error rate as covariate.
**Appendix S10** and **Table S5.** Whole‐brain results of the main effect of training in phase 2.
**Appendix S11.** Sensitivity analyses.
**Table S6.** Repeated‐measures ANCOVA of the behavioral data of phase 1 with IQ as covariate.
**Table S7.** Repeated‐measures ANCOVA of the behavioral data of phase 2 with IQ as covariate (Greenhouse–Geisser corrected).
**Table S8.** Repeated‐measures ANCOVA of the behavioral data of phase 3 with IQ as covariate.
**Table S9.** ANCOVA with the extracted beta values of the phase 2 group differences as dependent variables, group as independent variable and with IQ as covariate.
**Table S10.** ANCOVA with the extracted beta values of the phase 2 group differences as dependent variables, group as independent variable and with the slope of the phase 2 reaction times.
**Appendix S12.** gPPI seed selection and results.
**Figure S3.** Depiction of the 5 mm seed spheres for the gPPI analysis.
**Table S11.** 3DClustSim cluster size threshold results for each of the three gPPIs masked with the target regions.
**Figure S4.** Results of the gPPI analysis.

## Data Availability

The data that support the findings of this study are available on request from the corresponding author. The data are not publicly available due to privacy or ethical restrictions.
