# Supplementary material for: Avoidance habit learning in adolescents and young women with anorexia nervosa: an fMRI study
Source: J Child Psychol Psychiatry. 2025 Jul 31;67(1):79–91. doi: 10.1111/jcpp.70019 (PMC12699126; doi:10.1111/jcpp.70019)
Supplement: Supplementary file 1 — Appendix S1. Behavioral data quality control. Appendix S2. Data collection and exclusion criteria. Table S1. t‐Tests for the comparisons of the HCs included in current study with the ‘remaining’ HCs regarding demographic and clinical variables. Appendix S3. EMA procedure and data. Appendix S4. Experimental procedure (additional information). Appendix S5. Structural and functional image acquisition. Appendix S6. Functional image data processing and analysis. Appendix S7. gPPI analysis. Appendix S8 and Figure S1. Behavioral results of phase 1. Table S2. Repeated‐measures ANOVA of the behavioral data in phase 1. Appendix S9. Behavioral results of phase 3, strategies and sensitivity analysis. Figure S2. Behavioral results of phase 3. Table S3. Repeated‐measures ANOVA of the inverse efficiency score of the behavioral data of phase 3. Table S4. Repeated‐measures ANCOVA of the behavioral data of phase 3 with phase 1 mean error rate as covariate. Appendix S10 and Table S5. Whole‐brain results of the main effect of training in phase 2. Appendix S11. Sensitivity analyses. Table S6. Repeated‐measures ANCOVA of the behavioral data of phase 1 with IQ as covariate. Table S7. Repeated‐measures ANCOVA of the behavioral data of phase 2 with IQ as covariate (Greenhouse–Geisser corrected). Table S8. Repeated‐measures ANCOVA of the behavioral data of phase 3 with IQ as covariate. Table S9. ANCOVA with the extracted beta values of the phase 2 group differences as dependent variables, group as independent variable and with IQ as covariate. Table S10. ANCOVA with the extracted beta values of the phase 2 group differences as dependent variables, group as independent variable and with the slope of the phase 2 reaction times. Appendix S12. gPPI seed selection and results. Figure S3. Depiction of the 5 mm seed spheres for the gPPI analysis. Table S11. 3DClustSim cluster size threshold results for each of the three gPPIs masked with the target regions. Figure S4. Results of the gPPI analysis. [file JCPP-67-79-s001.docx]

Avoidance habit learning in adolescents and young women with anorexia nervosa: an fMRI study

***Supplementary Material***

Julius Hennig, Ilka Boehm, Katharina Zwosta, Joseph A. King, Daniel Geisler, Hannes Ruge, Maria Seidel, Fabio Bernardoni, Inger Hellerhoff, Arne Doose, Sophie Pauligk, Henri Leschzinski, Veit Roessner, Uta Wolfensteller, Stefan Ehrlich

[1. Behavioral data quality control 2](#_Toc187750724)

[2. Data collection, exclusion criteria and measures 2](#_Toc187750725)

[3. EMA procedure and data 4](#_Toc187750726)

[4. Experimental procedure (additional information) 5](#_Toc187750727)

[5. Structural and functional image acquisition 6](#_Toc187750728)

[6. Functional image data processing and analysis 6](#_Toc187750729)

[7. gPPI analysis 7](#_Toc187750730)

[8. Behavioral results of phase 1 8](#_Toc187750731)

[9. Behavioral results of phase 3, strategies and sensitivity analysis 9](#_Toc187750732)

[10. Whole-brain results of the main effect of training in phase 2. 11](#_Toc187750733)

[11. Sensitivity analyses of phase 2 fMRI results with IQ as covariate 12](#_Toc187750734)

[12. gPPI seed selection and results 13](#_Toc187750735)

[References 15](#_Toc187750736)

## Behavioral data quality control

Behavioral data underwent quality control prior to the statistical analyses based on the criteria used in Zwosta et al. (2018). Participants were excluded based on two criteria: (1) they showed an accuracy below 85% in block 7 of phase 2 (*M*=97.22%, *SD*=3.29%, range: 85%-100%) indicating failed habit induction; and (2) accuracy below 60% in the “no habit” condition of phase 3 (*M*=87.10%, *SD*=9.55%, range: 62%-100%) indicative of unclear instructions.

## Data collection, exclusion criteria and measures

The n=85 healthy control (HC) participants reported in the Methods section of the main manuscript were recruited as part of a larger study, which also includes an independent (yet-to-be analyzed) sample of weight-recovered individuals who no longer meet the diagnostic criteria for anorexia nervosa (AN). Because the two AN groups (usually younger acutely underweight and the somewhat older weight-recovered) partially overlap in age, there was a surplus of HC participants available for pairwise age-matching. This process was used to select the necessary number of HC participants to match the acute AN group, while the remaining (on average older) HC participants are reserved for inclusion in the weight-recovered cohort. The full number of HC participants is transparently reported in the participant flow to accurately reflect the recruitment process. Notably, the two HC samples only differed in age and the BIS score, and no other significant differences were evident (Table S1).

| **Table S1** | | |
| --- | --- | --- |
| *t-Tests for the comparisons of the HCs included in current study with the "remaining" HCs regarding demographic and clinical variables.* | | |
| measure | *t* | *p* |
| age | -5.67 | <.001 |
| BMI-SDS | 1.72 | .089 |
| parental SES | -0.63 | .531 |
| IQ | 1.37 | .173 |
| EDI-2 | -0.69 | .491 |
| BDI-II | -0.81 | .424 |
| SCL-90-R GSI | -1.78 | .083 |
| BSCS | -0.62 | .540 |
| BIS | -2.34 | .022 |
| BAS | -0.67 | .506 |
| *Note*. BMI-SDS=body mass index standard deviation score, SES=socioeconomic score, IQ=Intelligence Quotient, EDI-2=Eating Disorder Inventory-2, BDI-II=Beck's Depression Inventory-II, SCL-90-R GSI=Symptom Checklist-90-R Global Severity Index, BSCS=Brief Self-Control Scale, BIS=Behavioral Inhibition System, BAS=Behavioral Activation System. | | |

Clinical data were also collected from all participants using our own semi-structured research interview, which includes several specific questions to assess menstruation history, weight history, general medical and medication history, family psychiatric history, ethnicity, smoking status, and socioeconomic factors (e.g., educational level, occupation, family status, current living situation). Participants of both the acute AN and HC groups at the focus of the current analyses were excluded if they had a history of organic brain syndrome, schizophrenia, substance dependence, psychosis not otherwise specified (NOS), bipolar disorder, bulimia nervosa or binge-eating disorder. Further exclusion criteria were: age <12 years and age 30≤, IQ <85, psychotropic medication within 4 weeks prior to the study, current substance use disorder, inflammatory, neurologic or metabolic illness, chronic medical or neurological illness that could affect appetite, eating behavior, or body weight, clinically relevant anemia, pregnancy or breast feeding. HC were further excluded if they had a lifetime body mass index (BMI) below the tenth age percentile (if <18 yr)/BMI below 18.5kg/m² (if >18 yr), or were currently obese (BMI not over 97th age percentile if <18 yr; BMI not over 26kg/m² if >18 yr).

IQ was assessed with a short version of the Wechsler-Intelligence Scale for Children-IV (Petermann & Petermann, 2008) for participants aged 12-18 and of the German adaption of the Wechsler Adult Intelligence Scale (von Aster, Neubauer, & Horn, 2006) for participants age 18 and higher..

The socioeconomic status was calculated based on international standard occupational classification (Ganzeboom, De Graaf, & Treiman, 1992).

Inter-rater reliability of the interviews we used are 0.81 for current and 0.85 for past diagnoses measured by the SIAB-EX (Fichter & Quadflieg, 2001) and between 0.76 and 0.93 for the Mini-International Neuropsychiatric Interview (Lecrubier et al., 1997).

Internal consistency of the self-report measures: Cronbach’s alpha was 0.97 for the AN sample and 0.96 for the healthy control women for the sum score of the Eating Disorder Inventory-2 (Salbach-Andrae et al., 2010), 0.94-0.98 for the Global Symptom Index of the Symptom Checklist 90 Revised (Schmitz et al., 2000), 0.9 for the Beck Depression Inventory-II (Wang & Gorenstein, 2013), 0.91 for the Brief Self-Control Scale (Manapat, Edwards, MacKinnon, Poldrack, & Marsch, 2021) and 0.74 for the BIS subscale and 0.81 for the BAS subscale of the Behavioral Inhibition System/Behavioral Activation System (Strobel, Beauducel, Debener, & Brocke, 2001).

Notably, while the original BIS/BAS scale proposed a four-factor structure (three for BIS and one for BAS), the German translation has been validated with a two-factor structure, comprising one factor for BIS and one for BAS (Strobel et al., 2001).

Based on the study by Morean et al. (2014), we also calculated individual scores for the subscales “self-discipline” and “impulse control” of the BSCS for both groups. Testing group differences, we observed higher self-discipline values in the AN group compared to the HC group (AN: M=11.36, SD=2.12, HC: M=10.01, SD=2.04; t=2.84, p=.006, d=0.61), but not in impulse control (AN: M=12.44, SD=2.95, HC: M=13.0, SD=2.86; t=-0.86, p=.390, d=-0.18).

Study data were collected and managed using the secure, web-based electronic data capture tool REDCap (Research Electronic Data Capture; Harris et al., 2009).

## EMA procedure and data

At the beginning of the study, participants underwent a special EMA screening, were weighed, and were interviewed. They were also given detailed instructions on how to use the smartphone and the app, as in our previous studies (Fürtjes et al., 2020; Seidel et al., 2016). The sampling process began the day after the screening (for more details, see Seidel et al., 2022).

Participants in the study were asked if they had performed a habit in the past hour and in which category it belonged (e.g., food, hygiene, or public transport). The study focused on habits related to food intake and hygiene since the AN participants were mostly inpatients, limiting their daily routine. Participants were asked to provide details about each habit, such as the specific behavior for a hygiene habit.

For the EMA-related analyses, we only included datasets of participants with an EMA compliance rate >40%. Overall, the average EMA compliance for the AN group was 80.7% (*SD*=19.1%) and 69.0% (*SD*=23.9%) for the HC group. This difference was expected as HC participants had more limitations (e.g. school) to immediately answer prompts compared to the inpatient AN group.

Regarding the sample overlap between this study and the study by Seidel et al. (2022), we found that 59 of the 71 participants (83.1%) that had EMA (*n*=42 of 45 in the AN group, *n*=29 of 45 in the HC group) data were also included in the study by Seidel et al. (which in turn had a total of *n*=65 AN and *n*=89 HC participants).

## Experimental procedure (additional information)

*General task information:* The mappings of the stimuli categories were counterbalanced.

*Phase 1, establishing goal-directed behavior:* Responses were either a left or a right index finger response on the buttons “d” and “k” on a QUERTZ laptop keyboard. Phase 1 also had a practice phase with the instruction consisting of 12 trials, during which participants could ask clarifying questions. The present color of a cue was always the outcome color that was produced in the previous trial, or a random color in the first trial. The R‑O contingencies were preserved even in error trials. For example, if the task was to maintain a blue color but the incorrect response was made, an orange outcome color would appear instead.

*Phase 2, habit learning:* Negative outcomes (“-3” points) were presented in red and neutral outcomes (“0” points) in black.

## Structural and functional image acquisition

Structural and functional MRI data were acquired from all participants between 8 and 9 AM following an overnight fast with a 3T scanner (Magnetom Trio, Siemens) equipped with a standard head coil. The T1-weighted structural brain scans were acquired with rapid acquisition gradient echo (MP-RAGE) sequence with the following parameters: number of slices=176; repetition time (TR) 1900 ms; echo time (TE) 2.26 ms; flip angle 9°; slice thickness 1 mm; voxel size 1 × 1 × 1 mm³; field of view (FOV) 256 × 224 mm²; bandwidth 200 Hz/pixel. The functional images were acquired using gradient-echo T2*-weighted echo planar imaging (EPI) with the following parameters: tilted 17° from anterior–posterior commissure line toward coronal (to reduce signal dropout in orbitofrontal regions); number of volumes=435; number of slices=36; TR 2070 ms; TE 25 ms; flip angle 80°; 3 mm inplane resolution; slice thickness 3.2 mm (resulting in a voxel size of 3 × 3 × 3.2 mm³); FOV 192 × 192 mm²; bandwidth 2232 Hz/pixel.

## Functional image data processing and analysis

For fMRI preprocessing and analysis, we used SPM12 (http://www.fil.ion.ucl.ac.uk/spm/) unless otherwise specified. The functional images were corrected for temporal slice-timing and motion simultaneously using realign4D (Roche, 2011). The six realignment parameters, characterizing the rigid-body movement (x, y, z, pitch, roll, yaw), were saved and later used as nuisance covariates to account for the variance due to motion. Subsequently these images were coregistered to the participant’s structural brain image. A DARTEL template was created using structural images from all participants (Ashburner, 2007). The EPI volumes were then normalized to Montreal Neurological Institute space using the DARTEL template and corresponding flow field. The resulting data were smoothed with an isotropic 8 mm full-width at half-maximum Gaussian kernel. During the image data processing at the single participant-level using a general linear model (GLM), all regressors were convolved with a synthetic hemodynamic response function as implemented in SPM12.

## gPPI analysis

Prior to conducting the generalized psycho-physiological interaction (gPPI) analyses using the gPPI toolbox (McLaren, Ries, Xu, & Johnson, 2012), we used the denoising pipeline provided by the CONN toolbox (v21a, https://www.nitrc.org/projects/conn; Nieto-Castanon, 2021). Specifically, we utilized the functional artifact detection tool to add nuisance regressors, scrubbing, motion regression and temporal band-pass filtering to remove slowly fluctuating signals.

## Behavioral results of phase 1


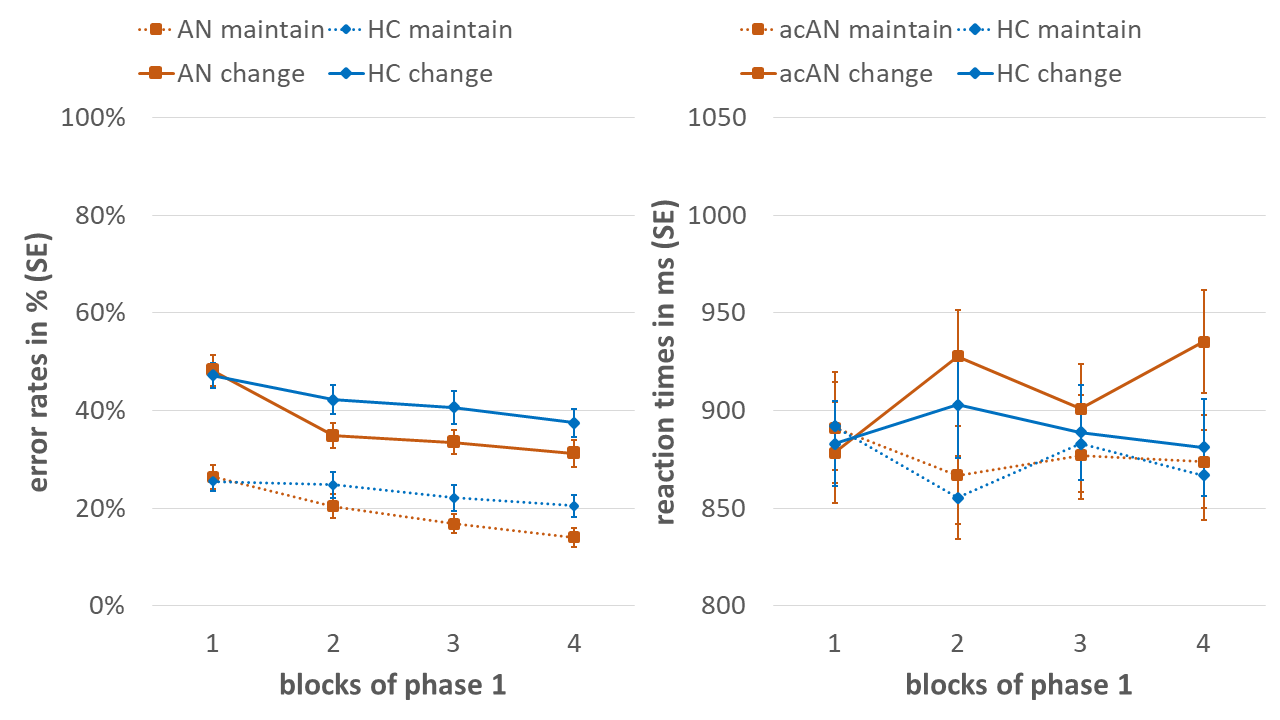


**Figure S1.** Behavioral results of phase 1. Error rates and reaction times over the course of phase 1, grouped into 4 blocks and separated for the acute anorexia nervosa (AN) and the healthy control (HC) group. Error bars depict the standard error (SE) of the mean.

| **Table S2** | | | | | | | | | |  |
| --- | --- | --- | --- | --- | --- | --- | --- | --- | --- | --- |
| *Repeated-measures ANOVA of the behavioral data of phase 1.* | | | | | | | | | |  |
|  | error rates | | | |  | reaction times | | | | |
|  | *df* | *F* | *p* | η_partial_ |  | *df* | *F* | *p* | η_partial_ | |
| condition | 1, 88 | 237.49 | <.001 | 0.730 |  | 1, 88 | 8.14 | .005 | 0.085 | |
| blocks | 3, 264 | 31.56 | <.001 | 0.197 |  | 3, 264 | 0.03 | .995 | <.001 | |
| group | 1, 88 | 2.73 | .102 | 0.030 |  | 1, 88 | 0.20 | .659 | 0.002 | |
| condition × blocks | 3, 264 | 2.00 | .114 | 0.022 |  | 3, 264 | 4.04 | .008 | 0.044 | |
| condition × group | 1, 88 | 0.24 | .628 | 0.003 |  | 1, 88 | 1.22 | .272 | 0.014 | |
| blocks × group | 3, 264 | 3.10 | .027 | 0.034 |  | 3, 264 | 0.75 | .524 | 0.008 | |
| condition × blocks × group | 3, 264 | 0.19 | .902 | 0.002 |  | 3, 264 | 0.56 | .642 | 0.006 | |

## Behavioral results of phase 3, strategies and sensitivity analysis


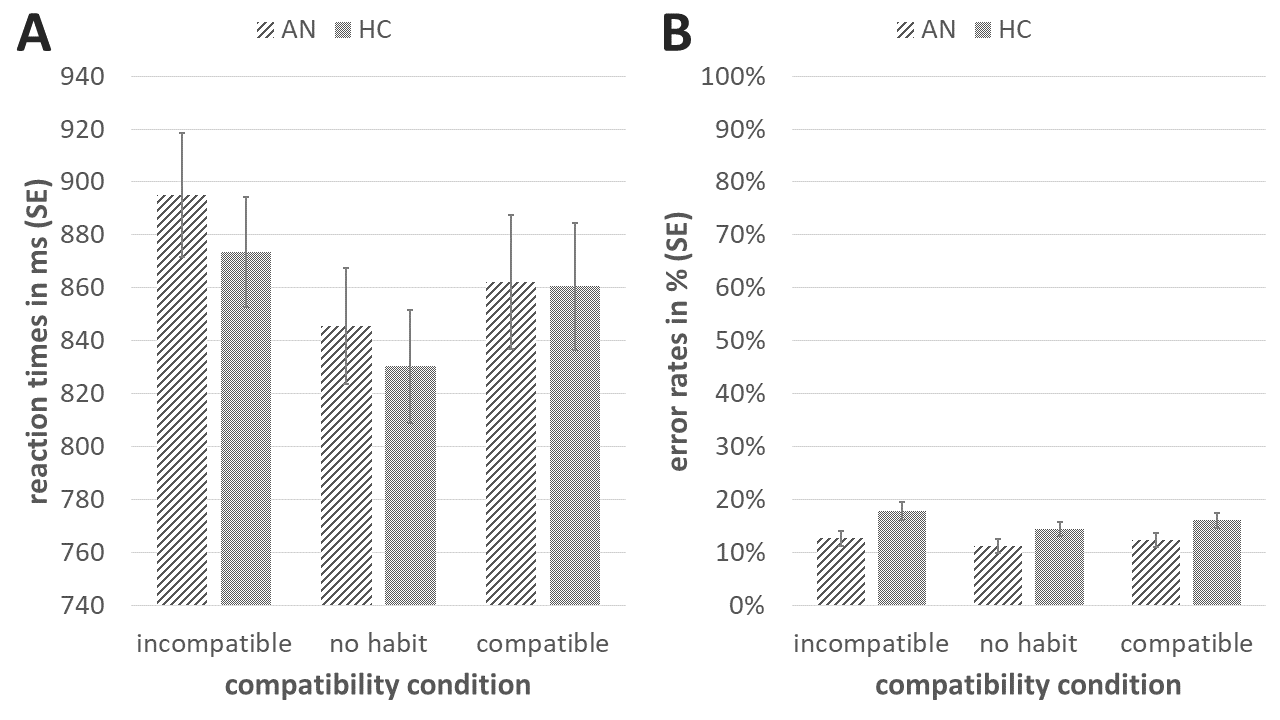


**Figure S2.** Behavioral performance of phase 3. Reaction times (A) and error rates (B) of the incompatible and the compatible as well as the no habit condition, separated for the acute anorexia nervosa (AN) and the healthy control (HC) group. Error bars depict the standard error (SE) of the mean.

To test whether different strategies were at play during phase 3, particularly with respect to a speed-accuracy trade-off in one of the groups, we additionally ran an ANOVA using the inverse efficiency score (IES) as dependent variable (Bruyer & Brysbaert, 2011). The IES has the advantage of simultaneously considering accuracy and speed by dividing reaction times with accuracy. In the ANOVA, we found that with respect to the IES, the performance in the compatible condition was significantly more optimal (i.e., lower IES) compared to the incompatible condition. Importantly, both groups showed similar strategies during phase 3.

| **Table S3** |  |  |  |  |
| --- | --- | --- | --- | --- |
| *Repeated-measures ANOVA of the inverse efficiency score of the behavioral data of phase 3.* | | | | |
|  | IES | | | |
|  | df | F | p | η_partial_ |
| compatibility | 1, 88 | 4.93 | .029 | 0.05 |
| group | 1, 88 | 0.60 | .441 | 0.01 |
| compatibility × group | 1, 88 | 0.002 | .965 | <0.01 |
| *Note.* IES = Inverse Efficiency Score. | | | | |

In order to investigate whether phase 1 performance, namely the mean error rate, influenced phase 3 performance, we conducted a sensitivity analysis by re-running the ANOVA described in the main manuscript as an ANCOVA.

Importantly, compared to the ANOVAs in the main manuscript, the results remain consistent for reaction times with a significant compatibility effect. The previously significant group effect in error rates only reached trend level. However, the crucial compatibility × group interaction remained non-significant. Expectedly, considering the similar rules/contingencies in phases 1 and 3, the covariate did reach significance for both behavioral metrics.

| **Table S4** |  | |  | |  | |  | |  |  | | |  | |  | |  | |  |
| --- | --- | --- | --- | --- | --- | --- | --- | --- | --- | --- | --- | --- | --- | --- | --- | --- | --- | --- | --- |
| *Repeated-measures ANCOVA of the behavioral data of phase 3 with phase 1 mean error rate as covariate.* | | | | | | | | | | | | | | | | | | |  |
|  | reaction times | | | | | | | | | error rates | | | | | | | | |  |
|  | | *df* | | *F* | | *p* | | η_partial_ | | |  | *df* | | *F* | | *p* | | η_partial_ | |
| compatibility | | 1, 87 | | 10.2 | | .002 | | 0.11 | | |  | 1, 87 | | 0.92 | | .341 | | 0.01 | |
| group | | 1, 87 | | 0.61 | | .439 | | <0.01 | | |  | 1, 87 | | 3.40 | | .069 | | 0.04 | |
| compatibility × group | | 1, 87 | | 2.65 | | .107 | | 0.03 | | |  | 1, 87 | | 0.45 | | .503 | | <0.01 | |
| phase 1 mean ER | | 1, 87 | | 5.87 | | .017 | | 0.06 | | |  | 1, 87 | | 13.1 | | <.001 | | 0.13 | |
| compatibility × phase 1 mean ER | | 1, 87 | | 2.45 | | .121 | | 0.03 | | |  | 1, 87 | | <0.01 | | .963 | | <0.01 | |

## Whole-brain results of the main effect of training in phase 2.

| **Table S5** | | | | | | | | | | | | |
| --- | --- | --- | --- | --- | --- | --- | --- | --- | --- | --- | --- | --- |
| Whole-brain results of the main effect of training in phase 2. FWE-corrected at *p*<.05. | | | | | | | | | | | | |
| direction | region of peak voxel | | peak voxel MNI coordinates | | | |  | | *T*_max_ | cluster size | | |
| decrease | Insula L | | -36 | -3 | 12 |  | | -10.41 | | | 13022 |  |
|  |  | Anterior insula L | -27 | 18 | 0 |  | | -9.6 | | |  |  |
|  |  | Precentral lobe L | -54 | 3 | 19 |  | | -8.88 | | |  |  |
|  | Triangular inferior frontal gyrus R | | 42 | 33 | 27 |  | | -5.40 | | | 132 |  |
|  | Cerebellum L | | -21 | -63 | -50 |  | | -4.66 | | | 91 |  |
|  |  | Cerebellum L | -36 | -66 | -54 |  | | -3.98 | | |  |  |
|  | Cerebellum R | | 18 | -66 | -50 |  | | -4.24 | | | 40 |  |
|  |  |  |  |  |  |  | |  | | |  |  |
| increase | Middle temporal pole R | | 48 | 12 | -35 |  | | 5.65 | | | 480 |  |
|  |  | Inferior temporal lobe R | 48 | 9 | -27 |  | | 5.12 | | |  |  |
|  |  | Superior temporal lobe R | 60 | -9 | 0 |  | | 5.01 | | |  |  |
|  | Middle temporal lobe L | | -60 | -3 | -27 |  | | 5.12 | | | 276 |  |
|  |  | Middle temporal lobe L | -60 | -6 | -8 |  | | 4.47 | | |  |  |
|  |  | Inferior temporal lobe L | -45 | 12 | -35 |  | | 4.47 | | |  |  |
|  | Gyrus rectus L | | -3 | 60 | -15 |  | | 5.01 | | | 133 |  |
|  | Medial prefrontal gyrus L | | -3 | 54 | 31 |  | | 4.81 | | | 261 |  |
|  |  | Middle frontal gyrus L | -27 | 30 | 50 |  | | 4.64 | | |  |  |
|  |  | Medial superior frontal gyrus L | -9 | 66 | 19 |  | | 4.44 | | |  |  |
|  | Angular gyrus L | | -54 | -57 | 35 |  | | 4.23 | | | 89 |  |
|  | Orbital inferior frontal gyrus L | | -39 | 27 | -19 |  | | 4.04 | | | 38 |  |
| *Note.* AAL atlas = automated anatomical labelling atlas, MNI coordinates = Montreal Neurological Institute coordinates, *T*_max_ = peak intensity t value. Positive *T*_max_ values represent a training-related linear increase of activity while negative *T*_max_ represent a decrease. | | | | | | | | | | | | |

## Sensitivity analyses

### **11.1 IQ group differences**

Given the significant group differences in IQ and the possibility that this might influence behavioral outcomes or group differences in the neural findings, we conducted sensitivity analyses using ANCOVAs. For the behavioral data, we included IQ as a covariate in the ANOVAs described in the main manuscript. For the neuroimaging results, we performed ANCOVAs with the extracted beta values of the phase 2 group differences as dependent variables, Group as dependent variable and IQ as covariate.

Importantly, the behavioral effects and interactions from all three phases were confirmed. While the effect of IQ itself was not significant in phases 2 and 3, it had a significant main effect on ER in phase 1, where we also observed a main effect of group on ER (Tables S6-S8).

| **Table S6** | | | | | | | | |
| --- | --- | --- | --- | --- | --- | --- | --- | --- |
| *Repeated-measures ANCOVA of the behavioral data of phase 1 with IQ as covariate.* | | | | | | | | |
|  | ER | | | |  | RT* | |  |
|  | *F* | *p* |  | *F* | | | *p* | |
| condition | 222.9 | <.001 |  | 11.6 | | | .001 | |
| blocks | 18.9 | <.001 |  | 0.06 | | | .968 | |
| group | 5.86 | .018 |  | 0.15 | | | .702 | |
| IQ | 7.75 | .007 |  | 0.22 | | | .643 | |
| condition × IQ | 4.07 | .047 |  | 1.54 | | | .218 | |
| condition × blocks | 1.88 | .133 |  | 3.70 | | | .016 | |
| condition × group | 1.72 | .193 |  | 3.66 | | | .059 | |
| blocks × IQ | 0.77 | .513 |  | 1.63 | | | .189 | |
| blocks × group | 3.15 | .026 |  | 0.61 | | | .586 | |
| condition × blocks × IQ | 1.44 | .232 |  | 1.60 | | | .195 | |
| condition × blocks × group | 0.60 | .618 |  | 1.07 | | | .357 | |
| *Note*. *Greenhouse-Geisser corrected. | | | | | | | | |

| **Table S7** |  | |  | |  | | |  | | | |  | |  | |  |  |  |
| --- | --- | --- | --- | --- | --- | --- | --- | --- | --- | --- | --- | --- | --- | --- | --- | --- | --- | --- |
| *Repeated-measures ANCOVA of the behavioral data of phase 2 with IQ as covariate (Greenhouse-Geisser corrected).* | | | | | | | | | | | | | | |  |  |  |  |
|  | RT | | | | | | | |  | ER | | |  |  |  |  |  |  |
|  | | *F* | | *p* | |  | *F* | | | | *p* | | | |  |  |  |  |
| training | | 44.4 | | <.001 | |  | 91.9 | | | | <.001 | | | |  |  |  |  |
| group | | <0.01 | | .977 | |  | 0.05 | | | | .816 | | | |  |  |  |  |
| training × group | | 0.73 | | .509 | |  | 0.74 | | | | .469 | | | |  |  |  |  |
| IQ | | 1.11 | | .295 | |  | 1.89 | | | | .173 | | | |  |  |  |  |
| training × IQ | | 0.64 | | .561 | |  | 1.35 | | | | .263 | | | |  |  |  |  |

| **Table S8** |  | |  | |  | | |  | | | | |  |  |  |  |  |
| --- | --- | --- | --- | --- | --- | --- | --- | --- | --- | --- | --- | --- | --- | --- | --- | --- | --- |
| *Repeated-measures ANCOVA of the behavioral data of phase 3 with IQ as covariate.* | | | | | | | | | | |  |  |  |  |  |  |  |
|  | RT | | | | | | | | ER | |  |  |  |  |  |  |  |
|  | | *F* | | *p* | |  | *F* | | | *p* | |  |  |  |  |  |  |
| compatibility | | 10.2 | | .002 | |  | 0.80 | | | .373 | |  |  |  |  |  |  |
| group | | <0.01 | | .985 | |  | 4.31 | | | .049 | |  |  |  |  |  |  |
| compatibility × group | | 0.29 | | .589 | |  | 0.41 | | | .522 | |  |  |  |  |  |  |
| IQ | | 1.27 | | .262 | |  | 0.29 | | | .591 | |  |  |  |  |  |  |
| compatibility × IQ | | 2.36 | | .128 | |  | 0.03 | | | .862 | |  |  |  |  |  |  |

Similarly, all four group differences of the phase 2 neuroimaging results remain, suggesting that the observed neural effects are robust and not driven by differences in IQ between the groups (Table S9).

| **Table S9** | | | |
| --- | --- | --- | --- |
| *ANCOVA with the extracted beta values of the phase 2 group differences as dependent variables, group as independent variable and with IQ as covariate.* | | | |
|  | effect | *F* | *p* |
| dmPFC | group | 17.6 | <.001 |
|  | IQ | 0.08 | .779 |
| IPL | group | 18.5 | <.001 |
|  | IQ | 0.04 | .850 |
| pMCC | group | 9.98 | .002 |
|  | IQ | 2.51 | .117 |
| pI | group | 16.3 | <.001 |
|  | IQ | 1.47 | .229 |
| Note. dmPFC=dorsomedial prefrontal cortex, IPL=inferior parietal lobe, pMCC=posterior mid-cingulate cortex, pI=posterior insula, IQ=intelligence quotient. | | | |

### **11.2 RT speeding**

RT speeding is commonly considered a characteristic of habitual responses (Seger & Spiering, 2011). However, it should not account for all the variance observed in BOLD signal changes during phase 2. This principle was demonstrated in the original study by Zwosta et al. (2018), which validated the task used in the current study.

For the present study, we conducted a sensitivity analysis using ANCOVA to test whether the group differences in the extracted beta mean values during phase 2 could be attributed to RT speeding. Specifically, we included individual RT slopes from phase 2 as a covariate in the ANCOVA. The results showed that the group differences in BOLD signal changes remained significant, while the RT slope did not reach significance (Table S10).

These findings suggest that the observed group differences in neural activity during phase 2 are unlikely to be fully attributable to RT speeding, aligning with the conclusions of Zwosta et al.

| **Table S10** | | | |
| --- | --- | --- | --- |
| *ANCOVA with the extracted beta values of the phase 2 group differences as dependent variables, group as independent variable and with the slope of the phase 2 reaction times.* | | | |
|  | effect | *F* | *p* |
| dmPFC | group | 25.59 | <.001 |
|  | p2 RT slope | 0.08 | .782 |
| IPL | group | 25.52 | <.001 |
|  | p2 RT slope | 1.24 | .269 |
| pMCC | group | 20.27 | <.001 |
|  | p2 RT slope | 1.14 | .289 |
| pI | group | 14.3 | <.001 |
|  | p2 RT slope | 1.43 | .236 |
| Note. dmPFC=dorsomedial prefrontal cortex, IPL=inferior parietal lobe, pMCC=posterior mid-cingulate cortex, pI=posterior insula. | | | |

## gPPI seed selection and results

For the gPPI analysis, we built 5mm spheres around the peak voxels of the training-related group differences of phase 2 in the dorsomedial prefrontal cortex (dmPFC) and the inferior parietal lobe (IPL) as part of the frontoparietal network (FPN), and the posterior midcingulate cortex (pMCC):


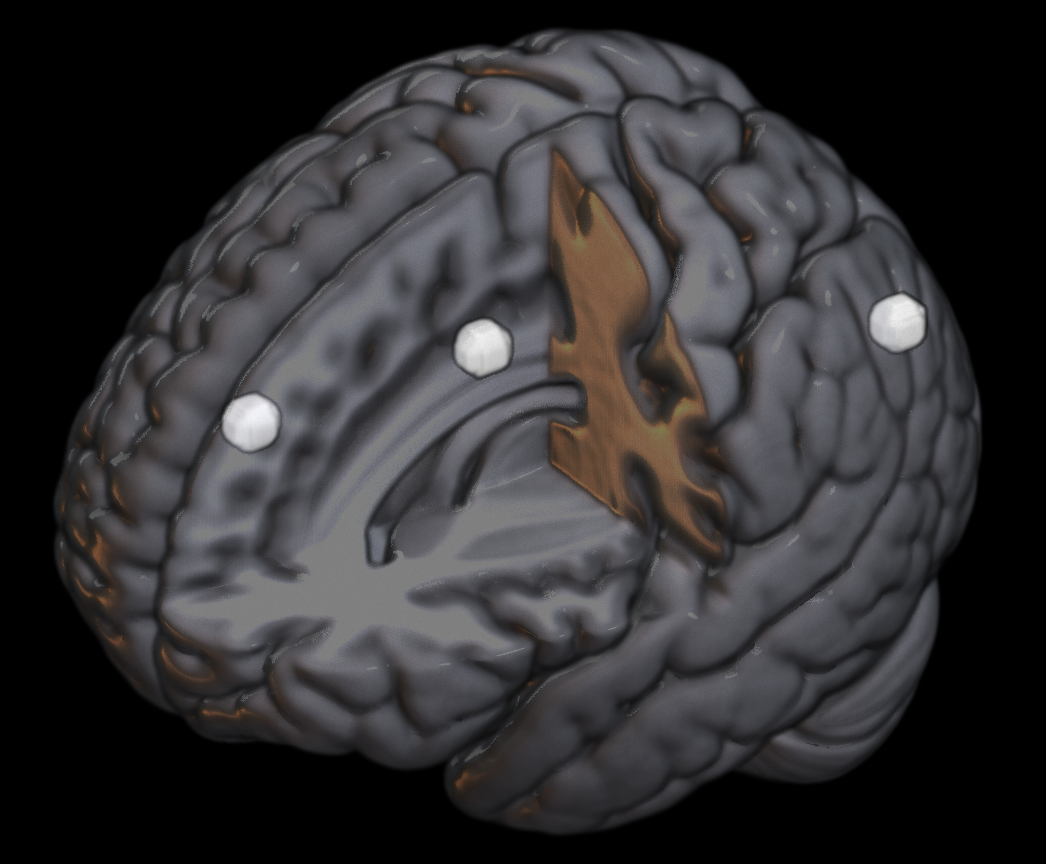


**Figure S3.** Depiction of the 5mm seed spheres for the gPPI analysis. From left to right: dorsomedial prefrontal cortex (center at x=-3, y=45, z=35), posterior midcingulate cortex (x=-9, y=-3, z=38), inferior parietal lobe (x=-51, y=-66, z=42).

For each seed, we then masked the gPPI results with the three remaining clusters of the task-related BOLD signal change group differences, e.g. dmPFC seed region to the pMCC, the posterior insula (pI) and the IPL as target regions (Figure S4).

Comparable to our whole-brain fMRI analyses, we used 3DClustSim (https://afni.nimh.nih.gov/, version from 24 Aug 2018) to control for false-positives. For the three gPPI analyses we conducted with seeds in the dmPFC, the IPL and the pMCC, we found the following cluster size thresholds (FWE-corrected *p*<.05):

| **Table S11** | | |
| --- | --- | --- |
| *3DClustSim cluster size threshold results for each of the three gPPIs masked with the target regions.* | | |
| seed region | target regions | *threshold* |
| dmPFC | IPL, pMCC, pI | 7.6 |
| IPL | dmPFC, pMCC, pI | 7.8 |
| pMCC | dmPFC, IPL, pI | 5.6 |
| *Note.* dmPFC=dorsomedial prefrontal cortex, IPL=inferior parietal lobe, pMCC=posterior mid-cingulate cortex, pI=posterior insula. | | |


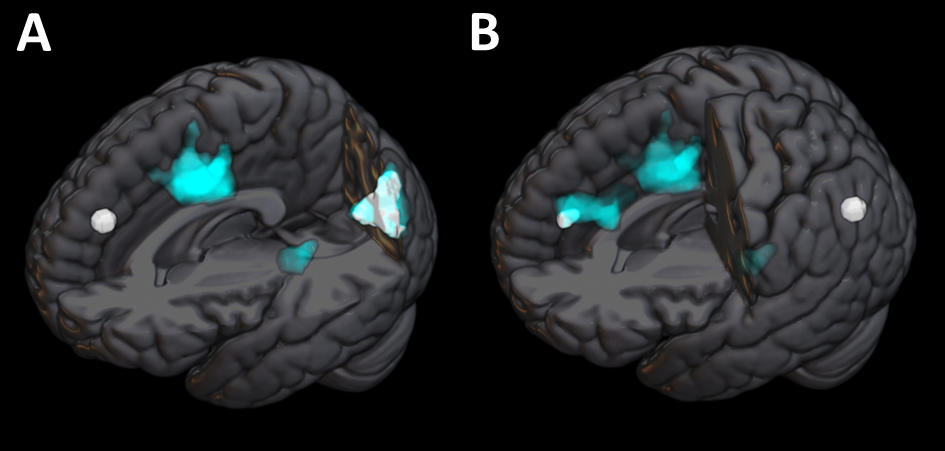


**Figure S4.** Results of the gPPI analysis showing training-related increase in functional connectivity (FC) during phase 2 in both groups. The seed spheres as well as the significant clusters are colored white while the masks are colored in blue. (A) gPPI results for the seed in the dorsomedial prefrontal cortex (dmPFC) masked with the phase 2 group differences of the task-related BOLD signal change in the posterior midcingulate cortex (pMCC), the posterior insula (pI) and the inferior parietal lobe (IPL), showing an FC increase in the IPL (*k*=122 voxels); (B) gPPI results for the seed in the IPL (masked with pI, pMCC and dmPFC), showing an FC increase in the dmPFC (*k*=10 voxels).

## References

Bruyer, R., & Brysbaert, M. (2011). Combining Speed and Accuracy in Cognitive Psychology: Is the Inverse Efficiency Score (IES) a Better Dependent Variable than the Mean Reaction Time (RT) and the Percentage Of Errors (PE)? *Psychologica Belgica*, *51*(1), 5. https://doi.org/10.5334/pb-51-1-5

Fichter, M., & Quadflieg, N. (2001). Das Strukturierte Interview für Anorektische und Bulimische Ess-Störungen nach DSM-IV und ICD-10 zur Expertenbeurteilung (SIAB-EX) und dazugehöriger Fragebogen zur Selbsteinschätzung (SIAB-S). *Verhaltenstherapie*, *11*(4), 314–325. https://doi.org/10.1159/000056675

Fürtjes, S., Seidel, M., King, J. A., Boehm, I., Jaite, C., Roessner, V., & Ehrlich, S. (2020). A naturalistic investigation of cognitive‐affective dysfunction in anorexia nervosa: The role of inefficiency. *International Journal of Eating Disorders*, *53*(2), 239–247. https://doi.org/10.1002/eat.23189

Ganzeboom, H. B. G., De Graaf, P. M., & Treiman, D. J. (1992). A standard international socio-economic index of occupational status. *Social Science Research*, *21*(1), 1–56. https://doi.org/10.1016/0049-089X(92)90017-B

Harris, P. A., Taylor, R., Thielke, R., Payne, J., Gonzalez, N., & Conde, J. G. (2009). Research electronic data capture (REDCap)—A metadata-driven methodology and workflow process for providing translational research informatics support. *Journal of Biomedical Informatics*, *42*(2), 377–381. https://doi.org/10.1016/j.jbi.2008.08.010

Lecrubier, Y., Sheehan, D., Weiller, E., Amorim, P., Bonora, I., Sheehan, K. H., … Dunbar, G. (1997). The Mini International Neuropsychiatric Interview (MINI). A short diagnostic structured interview: Reliability and validity according to the CIDI. *European Psychiatry*, *12*(5), 224–231. https://doi.org/10.1016/S0924-9338(97)83296-8

Manapat, P. D., Edwards, M. C., MacKinnon, D. P., Poldrack, R. A., & Marsch, L. A. (2021). A Psychometric Analysis of the Brief Self-Control Scale. *Assessment*, *28*(2), 395–412. https://doi.org/10.1177/1073191119890021

McLaren, D. G., Ries, M. L., Xu, G., & Johnson, S. C. (2012). A generalized form of context-dependent psychophysiological interactions (gPPI): A comparison to standard approaches. *NeuroImage*, *61*(4), 1277–1286. https://doi.org/10.1016/j.neuroimage.2012.03.068

Morean, M. E., DeMartini, K. S., Leeman, R. F., Pearlson, G. D., Anticevic, A., Krishnan-Sarin, S., … O’Malley, S. S. (2014). Psychometrically improved, abbreviated versions of three classic measures of impulsivity and self-control. *Psychological Assessment*, *26*(3), 1003–1020. https://doi.org/10.1037/pas0000003

Nieto-Castanon, A. (2021). *CONN functional connectivity toolbox (RRID:SCR_009550), Version 21*. Hilbert Press. https://doi.org/10.56441/hilbertpress.2161.7292

Petermann, F., & Petermann, U. (2008). *Hamburg Wechsler Intelligenztest für Kinder IV (HAWIK-IV)*. Bern: Huber.

Roche, A. (2011). A four-dimensional registration algorithm with application to joint correction of motion and slice timing in fMRI. *IEEE Transactions on Medical Imaging*, *30*(8), 1546–1554. https://doi.org/10.1109/TMI.2011.2131152

Salbach-Andrae, H., Schneider, N., Bürger, A., Pfeiffer, E., Lehmkuhl, U., & Holzhausen, M. (2010). Psychometrische Gütekriterien des Eating Disorder Inventory (EDI-2) bei Jugendlichen. *Zeitschrift für Kinder- und Jugendpsychiatrie und Psychotherapie*, *38*(3), 219–228. https://doi.org/10.1024/1422-4917/a000035

Schmitz, N., Hartkamp, N., Kiuse, J., Franke, G. H., Reister, G., & Tress, W. (2000). The Symptom Check-List-90-R (SCL-90-R): A German validation study. *Quality of Life Research*, *9*(2), 185–193. https://doi.org/10.1023/A:1008931926181

Seger, C. A., & Spiering, B. J. (2011). A Critical Review of Habit Learning and the Basal Ganglia. *Frontiers in Systems Neuroscience*, *5*. https://doi.org/10.3389/fnsys.2011.00066

Seidel, M., King, J. A., Fürtjes, S., Labitzke, N., Wronski, M.-L., Boehm, I., … Ehrlich, S. (2022). Increased Habit Frequency in the Daily Lives of Patients with Acute Anorexia Nervosa. *Nutrients*, *14*(19), 3905. https://doi.org/10.3390/nu14193905

Seidel, M., Petermann, J., Diestel, S., Ritschel, F., Boehm, I., King, J. A., … Ehrlich, S. (2016). A naturalistic examination of negative affect and disorder-related rumination in anorexia nervosa. *European Child & Adolescent Psychiatry*, *25*(11), 1207–1216. https://doi.org/10/gfrhfz

Strobel, A., Beauducel, A., Debener, S., & Brocke, B. (2001). Eine deutschsprachige Version des BIS/BAS-Fragebogens von Carver und White. *Zeitschrift für Differentielle und Diagnostische Psychologie*, *22*(3), 216–227. https://doi.org/10.1024//0170-1789.22.3.216

von Aster, M. G., Neubauer, A. C., & Horn, R. (2006). *WIE - Wechsler Intelligenztest für Erwachsene*. Bern: Huber.

Wang, Y.-P., & Gorenstein, C. (2013). Psychometric properties of the Beck Depression Inventory-II: A comprehensive review. *Revista Brasileira de Psiquiatria*, *35*(4), 416–431. https://doi.org/10.1590/1516-4446-2012-1048

Zwosta, K., Ruge, H., Goschke, T., & Wolfensteller, U. (2018). Habit strength is predicted by activity dynamics in goal-directed brain systems during training. *NeuroImage*, *165*, 125–137. https://doi.org/10.1016/j.neuroimage.2017.09.062
